# Methylenetetrahydrofolate Reductase (*MTHFR*) C677T Polymorphism and Subacute Combined Degeneration: Revealing a Genetic Predisposition

**DOI:** 10.3389/fneur.2018.01162

**Published:** 2019-01-09

**Authors:** Xin Zhang, Chen Hou, Peng Liu, Li Chen, Yue Liu, Peng Tang, Rui Li

**Affiliations:** ^1^Department of Neurology, Shaanxi Provincial People's Hospital, Xi'an, China; ^2^Department of Neurology, The Third Affiliated Hospital of Xi'an Jiaotong University School of Medicine, Xi'an, China

**Keywords:** subacute combined degeneration, methylenetetrahydrofolate reductase gene, homocysteine, vitamin B12, methionine

## Abstract

Vitamin B12 deficiency is regarded as the prevailing cause of subacute combined degeneration of the spinal cord (SCD). Nevertheless, the genetic predisposition to SCD remains unclear. The aim of this study was to explore the association between methylenetetrahydrofolate reductase gene (*MTHFR*) C677T polymorphism and SCD. We investigated *MTHFR* C677T polymorphism in SCD patients and found that the distribution of *MTHFR* C677T genotypes was significantly different between SCD patients and age-matched controls. Furthermore, the T allele frequency was markedly increased in SCD compared with the controls. In addition, the plasma homocysteine concentrations in subjects with the TT genotype were significantly elevated compared to those with the CC genotype. Logistic regression analysis results revealed that the *MTHFR* C677T genotype (TT vs. CT and CC) and vitamin B12 deficiency were risk factors for SCD. Our findings indicate that the T allele of the *MTHFR* C677T confers a strong genetic predisposition to SCD and provide evidence of an association between *MTHFR* C677T polymorphism and SCD. These data reveal a potential mechanism underlying SCD.

## Introduction

Subacute combined degeneration of the spinal cord (SCD) is pathologically characterized by demyelination and degeneration that occurs predominantly in the posterior and lateral columns of the spinal cord and, in rare cases, demyelination of peripheral nerves and white matter in the brain (1, 2). Vitamin B12 deficiency, which is believed to be associated with inadequate dietary intake and gastritis-associated vitamin B12 malabsorption, has long been regarded as the underlying cause of SCD (3, 4). However, risk factors for SCD other than vitamin B12 deficiency have not yet been identified.

Methylation is required for the synthesis of myelin in the spinal cord (5). Hypomethylation due to B12 deficiency is hypothesized to inhibit the conversion of homocysteine to methionine and then to S-adenosyl methionine (SAM), ultimately affecting myelin synthesis. Elevated homocysteine levels are thought to lead to increased concentrations of S-adenosyl homocysteine (SAH), a feedback inhibitor of methylation reactions (6). In addition, 5-methyltetrahydrofolate (5-MTHF) together with methyl-vitamin-B12 act as cofactors in the synthesis of methionine from homocysteine. The enzyme 5,10-methylenetetrahydrofolate reductase (MTHFR) converts 5,10-methylenetetrahydrofolate (5,10-MTHF) to 5-MTHF (7). Since 5-MTHF provides the methyl group for the conversion of homocysteine to methionine, which is essential for nucleotide synthesis as well as genomic and nongenomic methylation (6, 8), *MTHFR* C677T polymorphism may be involved in the B12 deficiency-mediated inhibition of this process. Thus, the relationship between *MTHFR* C677T polymorphism and SCD needs to be explored.

In this study, we investigated associations among vitamin B12, folate, homocysteine, and *MTHFR* C677T polymorphism in SCD patients to identify risk factors for SCD in a Chinese population.

## Materials and Methods

### Subjects

Thirty-one patients with SCD were consecutively recruited from Shaanxi Provincial People's Hospital, a comprehensive medical center located in northwestern China, between June 2014 and December 2016. Patients were enrolled if they met all the following inclusion criteria: (1) symmetrical abnormal neurological symptoms related to the posterior and lateral columns of the spinal cord (impairment of proprioception or vibrations or paresthesia in the lower or upper extremities, with or without corticospinal signs such as spasticity, hyperreflexia, the Babinski sign, hyperreflexia, and ankle reflex); (2) at least one abnormal laboratory test (mean corpuscular volume, megaloblastic anemia, hyperhomocysteinemia, or low serum vitamin B12 levels); (3) typical imaging findings, including inverted V sign on spinal MRI or periventricular hyperintensity on cranial MRI; and (4) symptoms that were not well explained by another diagnosis.

Subjects were excluded if they presented with a severe active infection, other spinal cord diseases, serious liver or kidney abnormalities or were under treatment for a previous SCD diagnosis. In addition, patients with other neurological diseases that cause similar symptoms, such as spinocerebellar ataxia, myelitis, multiple sclerosis, and spondylosis, were excluded based on the conventional MRI and lumbar puncture findings.

The clinical characteristics of patients with SCD, including age, sex, and history of macrocytic anemia and atrophic gastritis, were recorded. Plasma homocysteine, folate and vitamin B12 concentrations were measured, and *MTHFR* C677T polymorphism was analyzed.

Eighty age- and sex-matched subjects without spinal cord disease or a history of complementary medicine treatments containing vitamin B12, B6, or folate for at least 3 months were included in the control group.

### Blood Sample Collection and DNA Extraction

Whole blood samples (3 mL) were collected by venipuncture from participants. The samples were collected into microtainer tubes containing EDTA by a trained nurse in the morning (8:00–10:00 a.m.) and then stored at 2–8°C for analysis and measurement within 24 h. Genomic DNA was extracted using a TIANamp Blood Genomic DNA kit (TIANGEN BIOTECH, Beijing, China) according to the manufacturer's instructions. First, 1 to 2.5 times the volume of buffer CL was added to the blood samples and centrifuged at 10,000 rpm (~11,500 × g) for 1 min. Then, the supernatant was removed, 200 μL of buffer GS was added, and the mixture was vortexed. Then, 20 μL of proteinase K and 200 μL of buffer GB were added, and the mixture was incubated at 56°C for 10 min with shaking. A 200 μL volume of anhydrous ethanol was added, causing the DNA to precipitate. We placed the precipitated DNA and the solution into a CB3 column, centrifuged the column at 12,000 rpm (~13,400 × g) for 30 s, and discarded the flow-through. Then, 500 μL of GD buffer was added to the CB3 column, and the column was centrifuged at 12,000 rpm (~13,400 × g) for 30 s. The flow-through was then discarded. Next, we added 600 μL of buffer PW to the adsorbing CB3 column and centrifuged the column at 12,000 rpm (~13,400 × g) for 30 s. The solution was then centrifuged at 12,000 rpm (~13,400 × g) for 2 min. Finally, the CB3 column was placed into a 1.5 ml centrifuge tube, 50–200 μL of TB elution buffer was added, and the solution was incubated at room temperature for 2–5 min. Then, the mixture was centrifuged at 12,000 rpm (~13,400 × g) for 2 min, and the flow-through was transferred to a new centrifuge tube and stored at −20°C until subsequent analysis. The purity of the DNA was 1.7–1.9.

### Detection of the *MTHFR* C677T Polymorphism

PCR was performed using a fluorescent probe to detect the *MTHFR* C677T polymorphism. First, genomic DNA was extracted as described above. A total of 10 μL of the PCR reaction mixture (Taq DNA polymerase, PCR buffer, dNTPs, and MgCl_2_), 0.25 μL each of the probe and primers and 5.75 μL of double-distilled water were added to a centrifuge tube. Then, 4 μL of genomic DNA was added. The following primer sequences were used for amplification of the MTHFR gene: 5′-TGAAGGAGAAGGTGTCTGCGGGA-3′ (forward) and 5′ -AGGACGGTGCGGTGAGAGTG-3′ (reverse). The genotyping procedure was conducted according to the manufacturer's instructions (AUSA Pharmed Co, Ltd., Shenzhen, China). The following PCR protocol was used: one predenaturation step for 2 min at 50°C, followed by 45 cycles of a denaturation step at 95°C for 2 min, a reaction step at 95°C for 15 s, and a polymerization step at 60°C for 1 min. The reactions were performed in an Applied Biosystems 7500 instrument (Applied Biosystems, USA). After PCR amplification, the fluorescence signal at 60°C was used to analyze the *MTHFR* C677T polymorphism. The X-axis represented the “VIC signal” (C allele), and the Y-axis represented the “FAM signal” (T allele). When interpreting the results, the *MTHFR* C677T polymorphism was categorized as the CC (allele X), TT (allele Y), or CT (both alleles) genotype.

### Measurement of Plasma Homocysteine Concentrations

Plasma homocysteine concentrations were determined using a circulating enzymatic method according to the manufacturer's instructions (Purebio Biotechnology Co, Ltd., Ningbo, China) with a Hitachi 7170A analyzer (Hitachi Limited, Japan). The homocysteine detection reagents contained reagent R1 [Good's buffer (50 mmol/L), S-adenosylmethionine (0.1 mmol/L), NADH (0.2 mmol/L), ketoglutaric acid (5.0 mmol/L)], reagent R2 (R2- glutamate dehydrogenase (10 kU/L), S-adenosyl-L-homocysteine hydrolase (3 kU/L), adenosine deaminase (5 kU/L), homocysteine**-**transmethylase (5 kU/L)) and homocysteine calibration. The following parameters were used: the velocity method reaction type at a 340 nm wavelength and 37°C. First, 16 μL of the sample and 250 μL of R1 were combined for 3–5 min. Then, 25 μL of R2 was added for 90 s. The results were assessed after 180 s.

### Detection of Plasma Vitamin B12 Concentrations

Plasma vitamin B12 concentrations were detected with the chemiluminescence method according to the manufacturer's instructions (Beckman Coulter Inc., Brea, USA). Whole blood samples were collected by venipuncture into tubes containing heparin by a trained nurse in the morning (8:00–10:00 a.m.) after an overnight fast. They were then centrifuged and stored in a sealed tube at room temperature for <8 h. The specimens were detected using a Unicel Dxl 800 Access Immunoassay System (Beckman Coulter Inc., Brea, USA).

### Detection of Plasma Folate Concentrations

The competition combined with receptor method was used to detect plasma folate concentrations according to the manufacturer's instructions (Beckman Coulter Inc.). Whole blood samples were collected by venipuncture into tubes containing heparin by a trained nurse in the morning (8:00–10:00 a.m.) after an overnight fast. The specimens were centrifuged, and the plasma samples were then transferred to airtight tubes. The results were detected using a Unicel Dxl 800 Access Immunoassay System (Beckman Coulter Inc., Brea, USA). The plasma samples were stored at 2–8°C for no more than 8 h if they could not be immediately analyzed.

### Statistical Analysis

The ages of the participants are expressed as the mean ± SD. A *t*-test and one-way ANOVA were used to compare the difference of the two age-matched groups and three-genotype groups, respectively. The chi-square test was used to analyze *MTHFR* C677T polymorphism and categorical data between the control and SCD groups. Because the plasma homocysteine, vitamin B12 and folate concentrations exhibited abnormal distributions, we used their medians to analyze differences between the groups. The K-W H-test was used to compare plasma homocysteine concentrations among the three genotypes, and the Mann-Whitney U-test and Bonferroni's test were used to compare values between pairs of genotypes. Correlations between vitamin B12 and homocysteine concentrations were analyzed using Spearman's rho test. A logistic regression analysis was used to estimate odds ratios (ORs) with 95% confidence intervals (95% CIs) to identify risk factors of SCD. A *P*-value < 0.05 was considered statistically significant. All statistical analyses were performed using the International Business Machines (IBM) Statistical Package for the Social Sciences (SPSS) v. 21.0 for MAC OS.

## Results

### Demographic Data

The demographic data of the 111 participants are summarized in Table 1. The age of the patients with SCD ranged from 26 to 70 years old, and the age of the controls ranged from 32 to 80 years old. The median of the disease duration was 2.5 months (0.1 to 24 months). Among the 31 patients, 28 cases (90.3%), 26 cases (83.9%), 25 cases (80.6%), and 23 cases (74.2%) exhibited impairments in proprioception, vibrations, paresthesia, and corticospinal signs, respectively. A plasma vitamin B12 concentration < 180 pg/mL was defined as vitamin B12 deficiency, > 15 μmol/L as hyperhomocysteinemia, and < 3.1 ng/mL as folate deficiency. No differences were found in age, sex, the rates of history of atrophic gastritis or plasma folate deficiency between the SCD and control groups (*P* = 0.113, *P* = 0.434, *P* = 0.758, and *P* = 0.992, respectively). Significant differences were found in the history of macrocytic anemia and vitamin B12 deficiency between the two groups (*P* < 0.001 and *P* = 0.010, respectively). The median plasma homocysteine concentration was significantly higher in the SCD group (63.60 μmol/L) than in the control group (18.11 μmol/L) (*P* < 0.001).

**Table 1:** Characteristics of the study subjects.

**Parameters**	**Control** **(*N* = 80)**	**SCD** **(*N* = 31)**	**Z/χ^2^**	***P* value**
Age (years, mean ± SD)	61.38 ±9.97	57.77 ±12.26	1.599	0.113
Sex (*N*, M/F)	56/24	24/7	0.611	0.434
Disease duration (months, median)	NA	2.5	–	–
History of macrocytic anemia, *N* (percent)	5 (6.3)	17 (54.8)	33.192	0.000
History of atrophic gastritis, *N* (percent)	31 (38.8)	13 (41.9)	0.095	0.758
Impairment of proprioception, *N* (percent)	NA	28 (90.3)	–	–
Impairment of vibrations, *N* (percent)	NA	26 (83.9)	–	–
Paresthesia, *N* (percent)	NA	25 (80.6)	–	–
Corticospinal signs, *N* (percent)	NA	23 (74.2)	–	–
Hcy (μmol/L, median)	18.11	63.6	428.000	0.000
B12 deficiency, *N* (percent)^a^	23 (28.8)	17 (54.8)	6.597	0.010
Folate deficiency, *N* (percent)^b^	7 (8.8)	2 (6.5)	<0.001	0.992

SCD, subacute combined degeneration of the spinal cord; Hcy, homocysteine; NA, not available; M, Male; F, female.

Variables with normal distributions are presented as the means ± standard deviations, and non-continuous variables are presented as numbers and percentages.

The plasma homocysteine concentration is presented as a median.

a A plasma vitamin B12 concentration < 180 pg/mL was defined as B12 deficiency.

b *A plasma folate concentration < 3.1 ng/mL was defined as folate deficiency*.

### *MTHFR* C677T Genotype Distribution and Allele Frequencies in the Two Groups

The distribution of *MTHFR* C677T genotypes was balanced according to the Hardy-Weinberg genetic equilibrium test (χ^2^ = 0.134; *P* = 0.935) and a significant difference was found between the SCD and control groups (*P* = 0.006). The frequencies of the TT, CT, and CC genotypes in the control group were 27.5, 48.8, and 23.8%, respectively, whereas in the SCD group, they were 54.8, 41.9, and 3.2%, respectively. The T allele frequency in SCD subjects was significantly higher than that in the controls (75.8 vs. 51.9%), while the C allele frequency was lower in the SCD subjects than in the controls (24.2 vs. 48.1%) (*P* = 0.001) (Table 2).

**Table 2:** *MTHFR* C677T genotype distributions and allele frequencies in the SCD and control groups.

	***MTHFR*** **C677T genotype**			**Allele**	
	**TT**	**CT**	**CC**	**T**	**C**
Control (N = 80)	22	39	19	83	77
	27.5%	48.8%	23.8%	51.9%	48.1%
SCD (N = 31)	17	13	1	47	15
	54.8%	41.9%	3.2%	75.8%	24.2%
*P*-value (χ^2^)	0.006			0.001	

SCD, subacute combined degeneration of the spinal cord; MTHFR, methylenetetrahydrofolate reductase gene.

*The distribution of the MTHFR C677T genotype was significantly different between the SCD and control groups (χ^2^ = 10.198, P = 0.006). The T allele was detected at a significantly higher frequency in subjects with SCD than in the controls, while the C allele was detected at a lower frequency in subjects with SCD than in the controls (χ^2^ = 10.546; P = 0.001)*.

### Characteristics of Subjects With Different *MTHFR* Genotypes

The detailed data of the three *MTHFR* genotype groups were summarized in Table 3. The number of subjects with the TT, CT, and CC genotypes was 39, 52, and 20, respectively. Age and sex were comparable among the three groups (*P* = 0.479, *P* = 0.569, respectively). The plasma B12, folate, and homocysteine concentrations were determined for all subjects in the three *MTHFR* genotype groups. The median plasma homocysteine concentration was 45.80 μmol/L, 23.92 μmol/L, and 12.16 μmol/L in the TT, CT, and CC groups, respectively. Statistical analyses revealed significant differences among the three genotypes (H = 22.656, *P* < 0.001), especially between the TT and CC groups (U = 129.000, *P* < 0.001). However, no differences were found among the three genotypes in plasma B12 and folate concentrations (*P* = 0.189 and *P* = 0.412, respectively).

**Table 3:** Characteristics of subjects with different *MTHFR* genotypes.

**Parameters**	***MTHFR*** **genotypes**			**Z/χ^2^/F**	**P value**
	**TT**	**CT**	**CC**		
N	39	52	20		
Age (years, mean± SD)	61.77 ± 12.13	59.08 ± 10.16	61.00 ± 9.23	0.741	0.479
Sex (M/F)	30/9	35/17	15/5	1.128	0.569
B12 (pg/mL, median)	192.00	273.00	318.50	3.333	0.189
Folate (ng/mL, median)	11.85	11.49	9.41	1.773	0.412
Hcy (μmol/L, median)	45.80	23.92	12.16	22.656	< 0.001

SCD, subacute combined degeneration of the spinal cord; MTHFR, methylenetetrahydrofolate reductase gene; Hcy, homocysteine.

Plasma B12, folate and homocysteine concentrations are presented as medians.

*Median plasma homocysteine concentrations were significantly different among the three genotypes in all study subjects (H = 22.656, P < 0.001) and in the pairwise comparison of the TT and CC groups (U = 129.000, P < 0.001). Age, sex and plasma B12 and folate concentrations were comparable among the three genotypes in all study subjects (P = 0.479, P = 0.569, P = 0.189, P = 0.412, respectively)*.

### Correlations Between Plasma Folate and Vitamin B12 Concentrations and Homocysteine Concentrations

**Figure 1:**
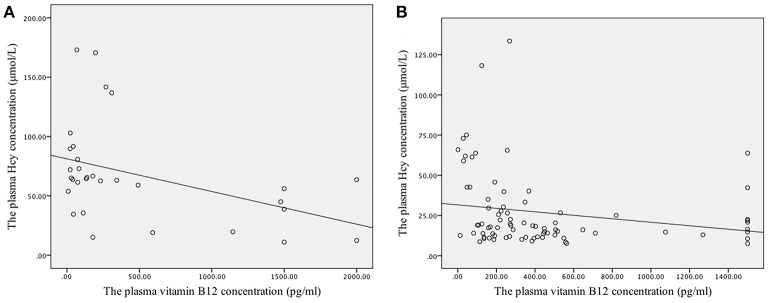
Correlation between the plasma concentrations of folate and vitamin B12 with homocysteine levels. **(A)** The linear correlation between homocysteine and vitamin B12 concentrations in the SCD group. Plasma homocysteine and vitamin B12 concentrations exhibited a negative linear correlation in the SCD group (r_s_ = −0.463, *P* = 0.009). **(B)** The linear correlation between homocysteine and vitamin B12 concentrations in the control group. Plasma homocysteine and vitamin B12 concentrations exhibited a negative linear correlation in the control group (r_s_ = −0.314, *P* = 0.005).

**Figure 2:**
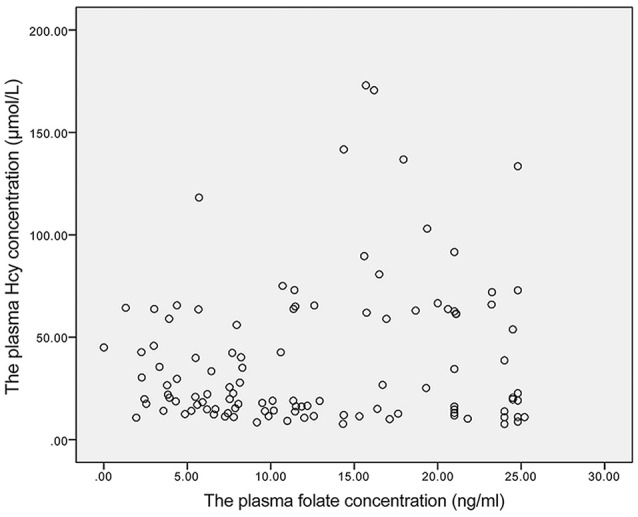
The linear correlation between homocysteine and folate concentrations. Homocysteine and folate concentrations did not exhibit a linear correlation (r_s_ = 0.009, *P* = 0.924).

A weak negative linear correlation was found between plasma homocysteine and vitamin B12 concentrations in all participants (r_s_ = −0.418, *P* < 0.001). Furthermore, in the SCD and control groups, a negative linear correlation was found between plasma homocysteine and vitamin B12 concentrations (r_s_ = −0.463, *P* = 0.009; r_s_ = −0.314, *P* = 0.005) (Figures 1A,B). However, no linear correlation was found between homocysteine and folate concentrations (r_s_ = 0.009, *P* = 0.924) (Figure 2).

### Adjusted ORs and 95% CIs for SCD

Potential risk factors for SCD, including the *MTHFR* C677T genotype (TT vs. CT and CC), vitamin B12 deficiency, folate deficiency, and hyperhomocysteinemia, were first analyzed with a single-factor logistic regression analysis. Among them, hyperhomocysteinemia was the most important factor associated with an increased risk of SCD (OR = 5.905, 95% CI 1.654 to 21.085, *P* = 0.006), followed by the *MTHFR* C677T genotype (TT vs. CT and CC, OR = 3.201, 95% CI 1.353 to 7.572, *P* = 0.008) and vitamin B12 deficiency (OR = 3.009, 95% CI 1.277 to 7.092, *P* = 0.012). However, folate deficiency did not increase the risk of SCD (OR = 0.719, 95% CI 0.141 to 3.668, *P* = 0.692). Furthermore, since homocysteine was correlated with both the *MTHFR* genotype and vitamin B12 concentration, we then performed a multifactor logistic regression analysis that was adjusted for the three mutually independent element factors, namely, the *MTHFR* genotype (TT vs. CT and CC), vitamin B12 deficiency and folate deficiency. The results showed that both *MTHFR* C677T genotype (TT vs. CT and CC) and vitamin B12 deficiency were positively correlated with SCD (OR = 2.882, 95% CI 1.189 to 6.986, *P* = 0.019 and OR = 2.742, 95% CI 1.129 to 6.662, *P* = 0.026, respectively). However, folate deficiency was still not associated with an increased risk of SCD (OR = 0.626, 95% CI 0.110 to 3.552, *P* = 0.597) (Table 4).

**Table 4:** ORs and 95% CIs for SCD.

	**Single-factor logistic regression**			**Multifactor logistic regression**		
	**OR**	**95% CI**	***P*-value**	**OR**	**95% CI**	***P*-value**
*MTHFR (TT vs. CT + CC)*	3.201	1.353–7.572	0.008	2.882	1.189–6.986	0.019
B12 deficiency^a^	3.009	1.277–7.092	0.012	2.742	1.129–6.662	0.026
Folate deficiency^b^	0.719	0.141–3.668	0.692	0.626	0.110–3.552	0.597
Hyperhomocysteinemia^c^	5.905	1.654–21.085	0.006	–	–	–

OR: odds ratio; 95% CI: 95% confidence interval.

a A plasma vitamin B12 concentration < 180 pg/mL was defined as B12 deficiency.

b A plasma folate concentration < 3.1 ng/mL was defined as folate deficiency.

c *A plasma homocysteic concentration >15 μmol/L was defined as hyperhomocysteinemia*.

## Discussion

SCD is a neurological complication caused by demyelination that occurs predominantly in the dorsal and lateral columns of the spinal cord (9). Its clinical manifestations include gait impairments, sensory abnormalities, ascending paresthesia and weakness in the extremities (10). Diagnosis of SCD is primarily based on clinical and laboratory tests and MRI observations showing hyperintense lesions in the posterior columns (11, 12). SCD was originally thought to be caused by vitamin B12 deficiency, which is typically associated with pernicious anemia (13), autoimmune gastritis, and insufficient dietary vitamin B12 intake (14). Although other possible risk factors, such as copper deficiency and nitrous oxide, were reported to be associated with SCD (14, 15), the genetic background remains unclear. In this study, we investigated plasma vitamin B12, folate, homocysteine concentrations, and *MTHFR* C677T polymorphism in SCD patients and identified the TT genotype of *MTHFR* C677T as a pivotal risk factor for SCD, thus revealing an important association between *MTHFR* C677T polymorphism and SCD.

Our results showed that patients with SCD had lower vitamin B12 levels and higher plasma homocysteine levels than the controls, which is consistent with the results of previous studies (4, 8, 16). We also found a negative linear correlation between plasma vitamin B12 and homocysteine levels in both the SCD and control groups, in agreement with the results of a study by Juan Ni et al. (17). Methylcobalamin and folate are cofactors involved in the methionine synthase-mediated conversion of homocysteine to methionine, a process that is essential for nucleotide synthesis and methylation (6). Therefore, a lack of methylcobalamin and folate would disrupt cell division and medullary myelin sheath synthesis and lead to homocysteine accumulation (8). This biochemical evidence may explain the vitamin B12 deficiency and increased homocysteine levels observed in patients with SCD, but the underlying mechanism must be explored further.

MTHFR, encoded by the *MTHFR* gene, is a key enzyme involved in the conversion of homocysteine to methionine. *MTHFR* also plays a vital role in DNA synthesis and methylation (18, 19). The most common polymorphism in the *MTHFR* gene is C677T (rs1801133). The enzymatic activity of the *MTHFR* T allele is lower than that of the C allele. As such, the *MTHFR* T allele is associated with reduced DNA methylation and increased homocysteine concentrations (20–23). Furthermore, *MTHFR* C677T polymorphism has been reported to be associated with various diseases such as birth defects, Alzheimer's disease, stroke, cancer, and psychiatric conditions (18, 19, 24, 25). A case report from Clayton et al. (16) showed that a 2-year-old girl with *MTHFR* gene deficiency developed autopsy-verified SCD, indicating that her *MTHFR* gene deficit might be related to SCD. However, to the best of our knowledge, no subsequent studies have explored the association between the *MTHFR* C677T polymorphism and SCD. Our study revealed that the frequency of the T allele in the *MTHFR* gene (C677T) was significantly increased in SCD patients compared to controls, indicating that the T allele of the *MTHFR* C677T confers a strong genetic predisposition for SCD.

Moreover, previous studies have demonstrated that the frequency of the *MTHFR* C677T polymorphism varies significantly among different ethnic groups (26). The prevalence of TT homozygosity ranges from 5 to 15% in Europe and North America, while it is 25% in China (27–29). A meta-analysis performed by Wang et al. (26) showed that the TT genotype and T allele frequencies in a healthy Chinese Han population were 22% (20–25%) and 45% (41–49%), respectively. Similarly, we found that the TT genotype and T allele frequencies in our control group were 27.5 and 51.9%, respectively. Moreover, *MTHFR* C677T polymorphism has been reported to vary along geographical gradients among Chinese populations—the frequencies of the 677TT genotype and the 677T allele increase along the southern-central-northern direction in mainland China, and the frequencies of the 677TT genotype and the 677T allele in northern China are 28% (25–31%) and 53% (51–55%), respectively (26). The population in our study was conducted in northern mainland China, a region in which the 677TT genotype and the 677T allele have a geographically high frequency.

The mechanisms by which *MTHFR* C677T polymorphism affects the pathogenesis of SCD remain to be elucidated. In our study, we found that plasma homocysteine concentrations were significantly different among the TT, CT, and CC genotypes, especially between the TT genotype group and CC genotype group, consistent with the results reported by Crider et al. (30). Thus, the increased risk of SCD observed in subjects carrying the T allele of the *MTHFR* gene could be due to impairments in homocysteine metabolism caused by reduced *MTHFR* activity. Homocysteine is metabolized by two major pathways: the remethylation pathway and the trans-sulfuration pathway. In the remethylation pathway, 5,10-MTHF is converted to 5-MTHF, which is the predominant form of folate in the blood; 5-MTHF subsequently acts as a methyl donor for homocysteine remethylation. MTHFR is a key enzyme for this irreversible conversion and plays a crucial role in controlling the distribution of folic acid throughout the metabolic pathway (31). Therefore, a reduction in MTHFR activity may decrease the concentration of 5-MTHF (17). The vitamin B12-dependent enzyme methionine synthase mediates the remethylation process, in which homocysteine is converted to methionine and then to SAM, a methyl donor for methylation reactions (6, 8). Therefore, a reduction in homocysteine-to-methionine remethylation would result in impaired nucleic acid metabolism and myelin sheath synthesis, which is closely associated with SCD (9). Because folate, vitamin B12 and MTHFR are all essential for the remethylation pathway, a deficiency in any one of these factors may affect the normal synthesis of methionine, leading to myelin sheath impairments and increased homocysteine concentrations (17).

Finally, we found that the *MTHFR* 677TT genotype, not vitamin B12, was the strongest risk factor for SCD. Since plasma homocysteine concentrations are determined by multiple factors, such as the *MTHFR* C677T genotype and vitamin B12 concentration, hyperhomocysteinemia may result from both the *MTHFR* 677TT genotype and vitamin B12 deficiency, which could be regarded as co-risk factors for SCD.

## Conclusion

These data demonstrate that *MTHFR* C677T polymorphism is strongly associated with SCD in a Chinese population, which underscores a genetic predisposition for SCD. The results of this study also suggest that *MTHFR* C677T polymorphism may affect myelin synthesis via methylation regulation, which is related to the pathogenesis of not only SCD but also many other conditions, including multiple sclerosis and myelin sheath dysplasia. Thus, identifying individuals with the *MTHFR* C677T polymorphism may provide guidance for novel therapeutic approaches to prevent the development of these diseases.

## Study Limitations

Our study has some limitations. First, as the low prevalence of SCD, the sample size was relatively small, which weakened the power of the findings. Therefore, further studies with larger sample sizes and a multicentered design need to be conducted. Second, since the distribution of *MTHFR* C677T polymorphism may differ by region, multi-region studies should be performed. Third, other potential risk factors for SCD, including other *MTHFR* polymorphisms, should be further studied. Finally, we did not investigate the relationship between *MTHFR* C677T polymorphism and other clinical aspects such as the prognosis and severity of neurological impairments, which need to be further elucidated.

## Ethics Statement

This study was carried out in accordance with the recommendations of the Ethics Committee of Shaanxi Provincial People's Hospital. The protocol was approved by the Ethics Committee of Shaanxi Provincial People's Hospital. All subjects gave written informed consent, and the study was performed in accordance with the Declaration of Helsinki.

## Author Contributions

XZ contributed to data acquisition, analysis and interpretation, manuscript composition, and study funding. CH contributed to data verification and analysis. PL contributed to data acquisition and analysis. LC contributed bibliographical services. YL contributed to manuscript drafting. PT contributed to data verification and table drafting. RL contributed to the study design and supervision, manuscript revision and analysis, and study funding.

### Conflict of Interest Statement

The authors declare that the research was conducted in the absence of any commercial or financial relationships that could be construed as a potential conflict of interest.
